# Automatic uncertainty evaluation for determining the number of components in nested models and the shrinkage regularization parameters

**DOI:** 10.1371/journal.pone.0354966

**Published:** 2026-08-14

**Authors:** Luca Martino, Roberto San Millán-Castillo, Eduardo Morgado

**Affiliations:** 1 Universitá degli studi di Catania, Catania, Italy; 2 Universidad Rey Juan Carlos, Fuenlabrada, Madrid, Spain; Institute of Theoretical and Applied Informatics Polish Academy of Sciences: Instytut Informatyki Teoretycznej i Stosowanej Polskiej Akademii Nauk, UKRAINE

## Abstract

In this study, we propose and examine two procedures for constructing intervals that capture the uncertainty associated with determining the effective number of components in model selection problems or the shrinkage parameters in a regularization problem. The output of these methods is an interval (defined by two integer bounds) representing plausible values for the number of components and/or shrinkage parameters. Notably, these methods do not rely on the availability of a likelihood function, making them broadly applicable across various domains, such as regression, classification, feature and/or order selection, clustering, and dimensionality reduction. These techniques leverage the geometric properties of the error curve to construct intervals. Extensive experiments on both synthetic and real-world datasets demonstrated the effectiveness and practical utility of the proposed procedures. In addition, a MATLAB code is provided to facilitate adoption by practitioners and researchers.

## 1. Introduction

Model selection is a fundamental task in contemporary signal processing, machine learning, and statistical analysis. See the following works [1–3] and [4] as examples showing the wide range of applications in different fields. Moreover, in recent decades, uncertainty quantification and sensitivity analysis have emerged as highly relevant research topics in various scientific fields [5–9]. Particularly important is the scenario of *nested models*, that is, a family of models of different complexities, where the number of parameters can grow (i.e., the dimension of the vector of parameters can grow, building more complex models). This scenario appears frequently in different real-world applications: for instance, The order selection in polynomial regression or autoregressive schemes [10,11], feature selection [12], clustering [13], change point detection [2,14], and dimension reduction are just a few [15,16]. Other relevant examples in signal processing are the estimation of the number of signal sources [17] and the so-called structured parameter selection [18,19]. Throughout this study, the terms variables, components, features, and parameters are used interchangeably to refer to the elements of a model.

In the literature, three primary classes of methods are commonly employed to infer the complexity of the nested models. The first class is formed by cross-validation (CV) techniques [20,21] or similar strategies [10,22]. The second class is the so-called probabilistic statistical measures, formed by two main subfamilies: the information criteria (IC) [13,23,24] and the marginal likelihood approach (a.k.a., Bayesian evidence) used in Bayesian inference [4,25,26]. Related schemes can also be found [27,28]. The third class comprises methods grounded in geometric considerations, such as automatic ’elbow’ [29–32] or ’knee-point’ detectors [33,34].

In [29], the authors demonstrated that the automatic elbow detectors proposed in the literature can be reformulated as a specific instance of an information criterion. Various information criteria (IC) differ in their choice of the slope parameter λ, which governs the penalization of model complexity [35], such as the Bayesian-Schwarz information criterion (BIC) and Akaike information criterion (AIC) (Table 1 provides different special cases of IC). Another recent approach, known as the Spectral information criterion (SIC), considers the entire spectrum of possible values for the penalization parameter λ, thereby encompassing other information criteria with linear complexity penalization as special cases. The SIC also returns a confidence measure of the proposed solution [36,37]. Similarly, other measures of reliability and confidence in the results in the context of elbow detection have been discussed [38]. These quantities attempt to show how ‘safe’ the solution is in terms of possible information lost by constructing a ‘too’ parsimonious model.

**Table 1:** Relevant examples of information criteria in the literature, with the corresponding choices of *V*(*k*) and λ. Note that *N* denotes the number of data points and $\ell_{\max}$ is the maximum value reached by the likelihood function.

Information criterion	Choice of λ	*V*(*k*)
Bayesian-Schwarz (BIC) [47]	logN	$-2\log\ell_{\max}$
Akaike (AIC) [48]	2	$-2\log\ell_{\max}$
Hannan-Quinn (HQIC) [49]	log(log(N))	$-2\log\ell_{\max}$
Universal Automatic Elbow Detector (UAED) [29]	*V*(0)/*K*	any
Spectral IC (SIC) [36]	all	any

The main contributions of this study are twofold: we extend one of the derivations proposed in [29] and the SIC derivation to provide an *interval* of indices corresponding to different possible models. Namely, the output of the proposed methods is an interval of possible number of components (defined by two specified integer values). This interval embeds the uncertainty associated with the decision in a black-box manner, contingent on the specific analysis and observed data. In the first proposed method, the underlying idea employs geometrical considerations, and it is inspired by the concept of maximum area under the curve (AUC) in receiver operating characteristic (ROC) curves [39,40] and by the derivation of the well-known Gini index [41–43]. We also show the relationship between the proposed procedure and an alternating optimization considering conditioned information criteria. The second proposed method employs the confidence measure provided by SIC to obtain an interval of possible models as the final solution. Moreover, we further extended the proposed techniques to the case where the error curve *V*(*z*) is defined over a continuous parameter *z*, and is observed at non-uniformly sampled points. This extension enables their use in selecting (providing an interval solution) the shrinkage regularization parameters, as in LASSO or ridge regularization problems [44,45]. The proposed schemes can also be applied in more general contexts than the standard IC, even when a probabilistic model is not considered and the likelihood function is not defined. Unlike the measures proposed in [38], the convexity of the error curve *V*(*k*) is not required in this study. Since the proposed methods identify reliable ranges of models rather than potentially risky single-point solutions, their range of applications is broad, encompassing areas such as the Internet of Things (IoT), smart cities, and effective governance, among many others [46]. Numerical experiments with artificial and real data showed promising results. Related Matlab code is also provided https://zenodo.org/records/21639880.

## 2. Framework and background

### 2.1. The error curve *V*(*k*)

In numerous real-world applications, we desire to infer a vector of parameters $\theta_{k}=[\theta_{1},...,\theta_{k}{]}^{⊤}$ of dimension *k* given a data vector $y=[y_{1},...,y_{N}{]}^{⊤}$. An observation model that induces a likelihood function $p(y|\theta_{k})$ is usually available [25]. The discrete variable *k*, which denotes the dimension of the parameter vector $\theta_{k}$, is often unknown and must be inferred from the observed data y. For example, *k* may represent the number of clusters in a clustering problem or the order of a polynomial in nonlinear regression task. In these application problems, a non-increasing error curve can be computed,


$$\begin{array}{c} V(k):\mathbb{N}\to \mathbb{R},\;k=0,1,2,...,K. \end{array}$$

where *K* represents the maximum possible dimension of the parameter vector, i.e., a model with *K* parameters, $\theta_{K}=[\theta_{1},...,\theta_{K}{]}^{⊤}$. The function *V*(*k*) can be any metric that characterizes system performance. For simplicity and without loss of generality, we consider an integer variable *k* with an increasing step of one unit. This can be easily generalized. when a likelihood function is given, a usual choice of *V*(*k*) is


$$\begin{array}{c} V(k)=-2\log(\ell_{\text{max}}),\;\text{ where }\;\ell_{\text{max}}=\max_{\theta_{k}}p(y|\theta_{k}), \end{array}$$

For example, as in [25,47,48]. Alternatively, *V*(*k*) can be directly defined as the mean square error (MSE), the mean absolute error (MAE), or transformations of them (e.g., as logMSE). However, any other fitting measures can be considered.

**Fig 1:**
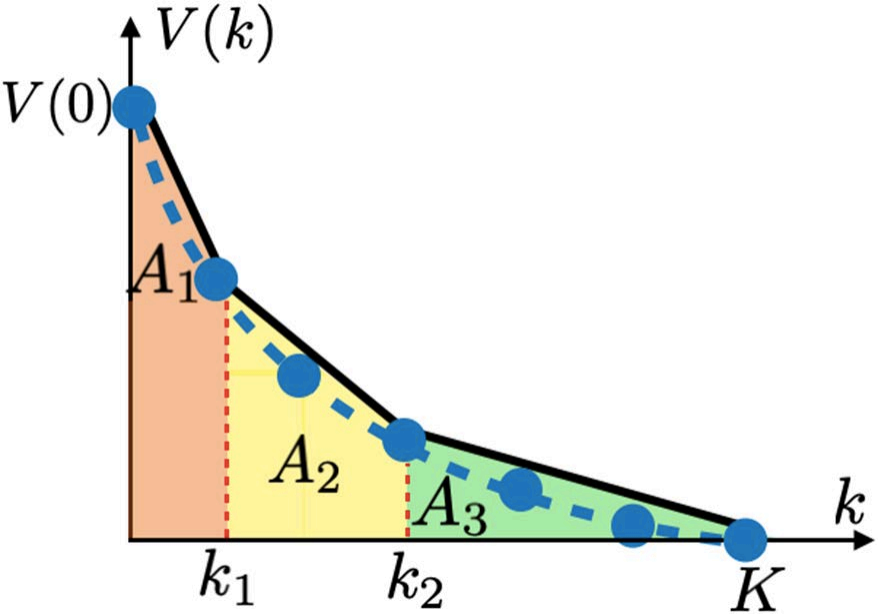
Example of error function *V*(*k*) (dashed line) and the construction of the areas *A*_1_, *A*_2_, *A*_3_, with three straight lines and $k_{1}<k_{2}$.

**Remark.** For the sake of simplicity and without loss of generality, we assume that minV(k)=V(K)=0. Clearly, this condition can always be obtained with a simple subtraction, $V^{\prime }(k)=V(k)-\min V(k)$. See Fig 1 for an example of *V*(*k*) (dashed line).

### 2.2. A general expression for several information criteria (IC)

**Fig 2:**
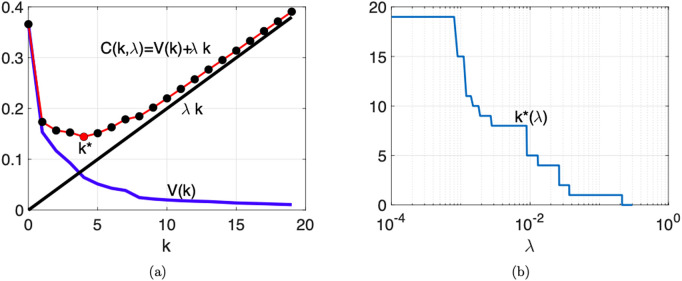
(a) Example of function *V*(*k*), the penalty λk (for a specific value of λ), and the resulting cost function C(k,λ)=V(k)+λk (shown with dots). **(b)** Example of a piecewise constant function $k^{*}(\lambda )$ yielded by SIC on a log-λ scale.

In this section, we outline a general formulation for various information criteria (IC) and the underlying principles of this approach. Typically, the curve *V*(*k*) is constructed as a non-increasing function. Graphical examples are shown in Figs 1 and 2(a). A well-established approach in the literature involves incorporating a linear penalty to account for model complexity,


$$\begin{array}{c} C(k)=C(k,\lambda )=V(k)+\lambda k,\;\lambda >0, \end{array}$$ (1)

where the slope of the complexity penalization term is denoted as λ. Since *V*(*k*) is non-increasing and the penalty term λk increases with respect to *k*, the cost function *C*(*k*) will exhibit at least one minimum. See Fig also 2(a), for a graphical example of *V*(*k*), the linear penalty λk and the corresponding cost function C(k)=C(k,λ).

The objective is to obtain $k^{*}=\text{arg}\min C(k)$ as the index of the *optimal* model, serving as an estimator for the number of components in the nested model. Clearly, the optimal value $k^{*}$ depends on the choice of λ. Table 1 summarizes some relevant special cases of IC, considering the possible choice of error curve *V*(*k*) and of the parameter λ. Some well-known examples are Bayesian-Schwarz information criterion (BIC), Akaike information criterion (AIC) and the Hannan-Quinn information criterion (HQIC), to name a few [47–49]. Each was derived in a distinct context and under different assumptions. In the literature, alternative IC with different analytical forms have been proposed (e.g., involving non-linear penalty terms); however, they are not as widely adopted as those with the form given in Eq. (1).

**Assumptions on *V*(*k*).** For clarity in the exposition, we have stated that *V*(*k*) is a non-increasing function. This assumption could even be relaxed, requiring only a decreasing trend, as in the (noisy) curves *V*(*k*) shown in Fig 5. Convexity is **not** a required condition. Regarding the geometric method in Section 3, the convexity of *V*(*k*) is a sufficient *but not necessary* condition for ensuring the uniqueness of the solution.

## 3. Interval solution based on geometric considerations

In this section, we present a novel technique for obtaining an interval of indices in the context of an elbow detection problem that encodes the uncertainty in model selection. More specifically, we extend one of the derivations of the universal automatic elbow detector, as presented in [29]. The underlying idea is inspired by the AUC approach in ROC curves for classification [39,40] and the derivation of the Gini index in the economic field [41,43].

### 3.1. Interval solution based on UAED approach

The algorithm is based on the construction of three straight lines: the first one passing through the points (0,*V*(0)) to $\left(k_{1},V(k_{1})\right)$; the second passes through the points $\left(k_{1},V(k_{1})\right)$ to $\left(k_{2},V(k_{2})\right)$ and the last passes through the points $\left(k_{2},V(k_{2})\right)$ to (*K*,0), as shown in Fig 1 (clearly, $k_{2}>k_{1}$). The goal is to minimize the area under this piece-linear approximation of the curve *V*(*k*). The total area under this approximation is the sum of the two trapezoidal areas (*A*_1_ and *A*_2_) and a triangular area (*A*_3_), as shown in Fig 1. Namely, we have


$$\begin{array}{cc} A_{1} & =\frac{(V(0)+V(k_{1}))k_{1}}{2},\\ A_{2} & =\frac{\left(V(k_{1})+V(k_{2}))(k_{2}-k_{1}\right)}{2},\;\text{ and }\\ A_{3} & =\frac{V(k_{2})(K-k_{2})}{2}, \end{array}$$

where we have used the assumption *V*(*K*)=0. Given the previous considerations, the optimal interval which possibly includes the location of an “elbow” point is defined as


$$\begin{array}{cc} \left[k_{1}^{*},k_{2}^{*}\right] & =\arg\;\min_{k_{1},k_{2}}J(k_{1},k_{2}),\\ & =\arg\;\min_{k_{1},k_{2}}\{A_{1}+A_{2}+A_{3}\},\\ & =\arg\;\min_{k_{1},k_{2}}\left\{k_{1}V(0)+k_{2}V(k_{1})-k_{1}V(k_{2})+KV(k_{2})\right\},\\ & =\arg\;\min_{k_{1},k_{2}}\left\{k_{1}V(0)+k_{2}V(k_{1})+(K-k_{1})V(k_{2})\right\},\;k_{1}<k_{2}. \end{array}$$ (2)

It is important to note that solving the optimization above is straightforward because *k*_1_ and *k*_2_ belong to a discrete and finite set. Note that by construction, the elbow $k_{\text{UAED}}$ provided by UAED in [29] is always contained in the obtained interval $\left[k_{1}^{*},k_{2}^{*}\right]$, i.e., $k_{\text{UAED}}\in [k_{1}^{*},k_{2}^{*}]$.

### 3.2. Relationship with the information criterion approach

If we keep fixed *k*_2_ as a constant, given Eq. (2), we have


$$\begin{array}{c} k_{1}^{*}=\text{arg}\min_{k_{1}}J(k_{1}|k_{2})=\text{arg}\min_{k_{1}}\left\{k_{1}V(0)+k_{2}V(k_{1})+(K-k_{1})V(k_{2})\right\}. \end{array}$$

We can rewrite it as a function of only *k*_1_ (given *k*_2_), i.e.,


$$\begin{array}{cc} k_{1}^{*} & =\text{arg}\min_{k_{1}}\left\{k_{2}V(k_{1})+(V(0)-V(k_{2}))k_{1}+KV(k_{2})\right\},\\ & =\text{arg}\min_{k_{1}}\left\{V(k_{1})+\frac{V(0)-V(k_{2})}{k_{2}}k_{1}\right\},\\ & =\text{arg}\min_{k_{1}}\left\{C(k_{1}|k_{2})\right\}, \end{array}$$ (3)

where we have used that *k*_2_ and *K* are considered constant. The last expression


$$\begin{array}{c} C(k_{1}|k_{2})=V(k_{1})+\lambda (k_{2})k_{1}, \end{array}$$ (4)

has the form of an information criterion where *V*(*k*_1_) plays the role of the error curve and the slope λ is here a function of *k*_2_, i.e.,


$$\begin{array}{c} \lambda (k_{2})=\frac{V(0)-V(k_{2})}{k_{2}}, \end{array}$$

instead of being constant. If *k*_2_ = *K* and *V*(*K*)=0, we recover the slope $\lambda =\frac{V(0)}{K}$ associated with the automatic elbow detector in [29], as shown in Table 1. Fixing *k*_1_ as a constant, given Eq. (2), we have


$$\begin{array}{c} k_{2}^{*}=\text{arg}\min_{k_{2}}J(k_{2}|k_{1})=\text{arg}\min_{k_{2}}\left\{k_{1}V(0)+k_{2}V(k_{1})+(K-k_{1})V(k_{2})\right\}, \end{array}$$

and we can rewrite it as a function of only *k*_2_ (given *k*_1_), i.e.,


$$\begin{array}{cc} k_{2}^{*} & =\text{arg}\min_{k_{2}}\left\{k_{2}V(k_{1})+(K-k_{1})V(k_{2})\right\}\\ & =\text{arg}\min_{k_{2}}\left\{V(k_{2})+\frac{V(k_{1})}{K-k_{1}}k_{2}\right\},\\ & =\text{arg}\min_{k_{2}}\left\{C(k_{2}|k_{1})\right\}, \end{array}$$ (5)

where we have used that *k*_1_ and *K* are constant, and set


$$\begin{array}{c} C(k_{1}|k_{2})=V(k_{2})+\lambda (k_{1})k_{2}, \end{array}$$ (6)

that, again, has the form of an information criterion where the slope λ is a function of *k*_1_, i.e.,


$$\begin{array}{c} \lambda (k_{1})=\frac{V(k_{1})}{K-k_{1}}, \end{array}$$

instead of a constant. If we set *k*_1_ = 0, we again recover the slope $\lambda =\frac{V(0)}{K}$ associated with the automatic elbow detector in [29]. Then, considering an alternating optimization approach, minimizing $J(k_{1},k_{2})=A_{1}+A_{2}+A_{3}$ can be interpreted as minimizing iteratively different conditioned information criteria, considering $J(k_{1}|k_{2})$ and $J(k_{2}|k_{1})$, which are connected to each other by the different slopes of the complexity penalty.

## 4. Interval solution based on SIC approach

### 4.1. Principles underlying the SIC technique

For simplicity, assume that *V*(*k*) is strictly decreasing, such that *C*(*k*) has a unique minimum. In [36], firstly the authors note that $\lambda \in [0,\lambda_{\text{max}}]$,


$$\begin{array}{c} \lambda_{\text{max}}=\max_{k}\left[\frac{V(0)-V(k)}{k}\right],\;\text{for }k=1,...,\text{K}. \end{array}$$ (7)

Indeed, for $\lambda \geq \lambda_{\text{max}}$ we always obtain $k^{*}=\text{arg}\min C(k)=0$. It is interesting to note the relationship between $\frac{V(0)-V(k)}{k}$ in Eq. (7) and $\lambda (k_{2})$ in Eq. (4). Generally, the position of the minimum changes $k^{*}$, by varying λ in $\left[0,\lambda_{\text{max}}\right]$. Namely, the location of the minimum a function of λ,


$$\begin{array}{c} k^{*}(\lambda )=k_{\text{min}}=\text{arg}\min_{k}C(k,\lambda ),\;k^{*}(\lambda ):[0,\lambda_{\text{max}}]\to \{0,1,2...,K\}. \end{array}$$ (8)

It is a non-increasing, piecewise constant function taking discrete values from 0 to *K*, where $k^{*}(0)=K$ and $k^{*}(\lambda )=0$ for $\lambda \geq \lambda_{\text{max}}$. It is a piecewise constant function since, even changing λ, the value $k^{*}(\lambda )$ can remain unvaried. See Fig 2(a) for an example of the function *V*(*k*) and the corresponding cost function C(k,λ) for a given value of λ. The resulting function $k^{*}(\lambda )$ is shown in Fig 2(b).

**Fig 3:**
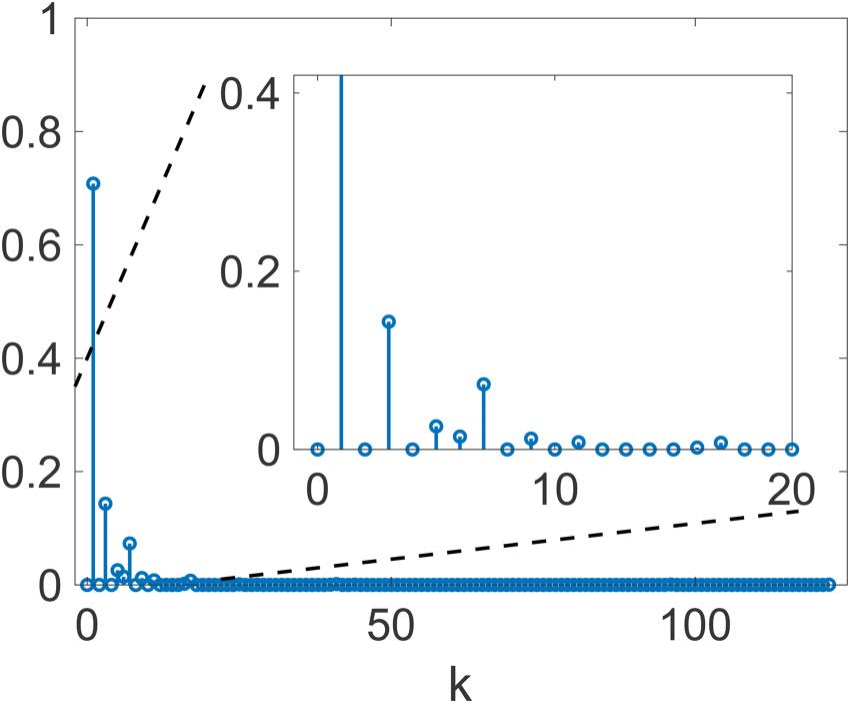
Example of SIC weights associated to the different possible minima of the cost function C(k,λ). The weights ${\bar{w}}_{k}$ obtained by the SIC method correspond to the (black) error curve *V*(*k*) in Figure 2(a), and are used in the experiment in Section 7.4. Note that 0≤k≤122, i.e., *K* = 122. It is important to emphasize that the number of nonzero weights is significantly smaller than 122.

As shown in Fig 2(b), different values of $\lambda^{\prime }$ and $\lambda^{\prime \prime }$ can yield the same minimum denoted as *j*, i.e., $k^{*}(\lambda^{\prime })=j$ and $k^{*}(\lambda^{\prime \prime })=j$, so that the function $k^{*}(\lambda )$ remains constant in certain pieces, each one characterized by a specific “length”. Thus, varying λ, to each possible solution denoted by an index {0,1,...,*K*}, we have associated a length value, i.e.,length of the constant piece; see Fig 2(b). We can convert these lengths into weights, associating to each index j∈{0,1,..,K} a normalized weight ${\bar{w}}_{j}$. This weight ${\bar{w}}_{j}$ is computed approximately in Eq. (10): it quantifies the robustness of the *j*-th minimum obtained in Eq. (8), with respect to variations in λ. In other words, a large weight ${\bar{w}}_{j}$ indicates that the model with *j* components appears as a solution of the minimization in (8) repeatedly across a wide range of λ values. Moreover, some solutions – say, for instance, with *k* components – may never appear for any value of λ; in that case, ${\bar{w}}_{k}$ must be zero, ${\bar{w}}_{k}=0$. Note that, by construction, we have ${\bar{w}}_{0}=0$ (by definition of $\lambda_{\text{max}}$). Fig 3 provides a graphical representation of this probability mass.

A Monte Carlo procedure to compute approximately these normalized weights ${\bar{w}}_{j}$ is given in Table 2, using $M\geq 10^{5}$ uniform samples from $𝒰([0,\lambda_{\text{max}}])$. A more efficient alternative to the Monte Carlo approach is to use a fine grid dividing the interval $\left[0,\lambda_{\text{max}}\right]$ in $M\geq 10^{5}$ uniform sub-intervals, which is the strategy implemented in the provided MATLAB code. The two approaches are equivalent. The Monte Carlo procedure is perhaps more illustrative; therefore, it is described in Table 2. In contrast, the fine-grid procedure is more efficient, as it exhibits lower variance because it is equivalent to a stratified/systematic sampling approach.

**Table 2:** Computation of the weights in the SIC method by Monte Carlo. Clearly, alternative quasi-Monte Carlo can be employed (using a fine grid of λ values).

• Choose a value *M* (e.g., $M\geq 10^{5}$) and calculate $\lambda_{\text{max}}=\max_{k}\left[\frac{V(0)-V(k)}{k}\right]$. • For *i* = 1,...,*M*: 1. Generate randomly $\lambda_{i}\sim 𝒰([0,\lambda_{\text{max}}])$. 2. Compute $k_{i}=\text{arg}\min_{k}C(k,\lambda_{i})=\text{arg}\min_{k}\left[V(k)+\lambda_{i}k\right].$ (9) • Return the frequency of the event $\left\{k_{i}=j\right\}$ for *j* = 1,...,*K*, that is equivalent to return the weights ${\bar{w}}_{j}=\frac{\#\{k_{i}=j\}}{M},\;j=0,...,K.$ (10)

Note that the normalized weights form a probability mass function (pmf). In order to design an interval $\left[k_{1}^{*},k_{2}^{*}\right]$ including the possible elbow of the curve (and encoding the related uncertainty), we define a cumulative sum of the first *k* weights, denoted as $W_{k}$, i.e.,


$$\begin{array}{c} W_{k}=\sum_{j=1}^{k}{\bar{w}}_{j}, \end{array}$$ (11)

with 0<k≤K.

### 4.2. Probability interpretations and interval solution

In this section, we provide additional theoretical support and interpretation for the quantities obtained through the SIC methodology. In particular, we discuss their statistical interpretation and the rationale underlying their choice and use. Choosing uniformly $\lambda^{\prime }\sim 𝒰([0,\lambda_{\text{max}}])$, each weight represents the probability that the number of components of the model is $k_{\text{min}}=j$, i.e.,


$$\begin{array}{c} {\bar{w}}_{j}=\text{Prob}\left\{k_{\text{min}}=j\right\},\;\text{ where }\;k_{\text{min}}=\text{arg}\min_{k}V(k)+\lambda^{\prime }k,\;\text{ and }\;\lambda^{\prime }\sim 𝒰([0,\lambda_{\text{max}}]). \end{array}$$ (12)

Fig 3 shows an example. Then, The cumulative distribution $W_{k}=\sum_{j=1}^{k}{\bar{w}}_{j}$ has the following probabilistic meaning:


$$\begin{array}{c} W_{k}=\text{Prob}\left\{k_{\text{min}}\leq k\right\},\;\text{ where }\;k_{\text{min}}=\text{arg}\min_{k}V(k)+\lambda^{\prime }k,\;\text{ and }\;\lambda^{\prime }\sim 𝒰([0,\lambda_{\text{max}}]). \end{array}$$ (13)

We note that sampling $\lambda^{\prime }$ uniformly from the interval $\left[0,\lambda_{\text{max}}\right]$ provides an objective and robust strategy, as it avoids favoring the selection of any particular model.

At the same time, however, we aim to avoid selecting *overly parsimonious* models (i.e., excessively simple models), which are typically associated with large values of the penalization parameter λ; see Fig 2(b). Note that $W_{k}$ can be interpreted as the a **detection probability**, i.e., the probability of finding a model that correctly describes the phenomenon (the true model, denoted as $k_{\text{true}}=k^{*}$). Therefore, increasing the probability $W_{k}$ – in order to reduce the risk of missing relevant components in the model— leads to a larger value of *k*. Several empirical studies reported in [36] indicate that threshold values around ℓ≈0.95 provide a high probability of correct detection. Then, we set the interval solution $\left[k_{1}^{*},k_{2}^{*}\right]$ where


$$\begin{array}{cc} k_{1}^{*} & =\min\{k:\;W_{k}\geq \ell_{1}\},\\ k_{2}^{*} & =\min\{k:\;W_{k}\geq \ell_{2}\},\;\ell_{1}<\ell_{2}. \end{array}$$ (14)

Note that $\ell_{1},\ell_{2}$ play a similar role to a confidence level. After several empirical studies, we can assert that a very robust choice is given by $\ell_{1}=0.95$ and $\ell_{2}=0.995$. Safer intervals can be designed by considering a more conservative value, such as $\ell_{2}=0.999$.

## 5. Interpreting the interval solutions

The methods described above return an interval $\left[k_{1}^{*},k_{2}^{*}\right]$ as the final solution. In this section, we first explain how to interpret the interval. We then applied both techniques to ideal scenarios to validate and illustrate the intuition underlying these interpretations.

### 5.1. Interpretation

The two interval solutions obtained by the UAED-geometric and SIC approaches have two different interpretations, which are described below. In both cases, the true number of components $k^{*}$ should be contained in the interval $\left[k_{1}^{*},k_{2}^{*}\right]$, that is, $k^{*}\in [k_{1}^{*},k_{2}^{*}]$. In both cases, Hence, shorter SIC intervals imply that we are more certain that $k^{*}\in [k_{1}^{*},k_{2}^{*}]$. However, the principles underlying the construction of the interval are different.

- the SIC-based interval is build to provide an interval where with high probability is contained the true model $k^{*}$, i.e., in some sense, maximizing the probability $\text{Prob}\left(k^{*}\in [k_{1}^{*},k_{2}^{*}]\right)$. Since $W_{k}$ represents a detection probability, the interval defines a range of solutions that reduces the risk of overlooking relevant components in the model.
- The UAED-geometric interval is constructed according to a more robust principle: ensuring that the elbow point is not contained in the intervals $\left[0,k_{1}^{*}\right)$ and $\left(k_{2}^{*},K\right]$. In other words, the UAED-geometric interval can be viewed as being designed to try to maximize the probability $\text{Prob}\left(k^{*}\notin [0,k_{1}^{*})\text{ and }k^{*}\notin (k_{2}^{*},K]\right)$.

Therefore, due to these two different construction philosophies, it is intuitively clear that the UAED-geometric intervals are generally wider than the corresponding SIC-based intervals.

### 5.2. Application to ideal scenarios

**Fig 4:**
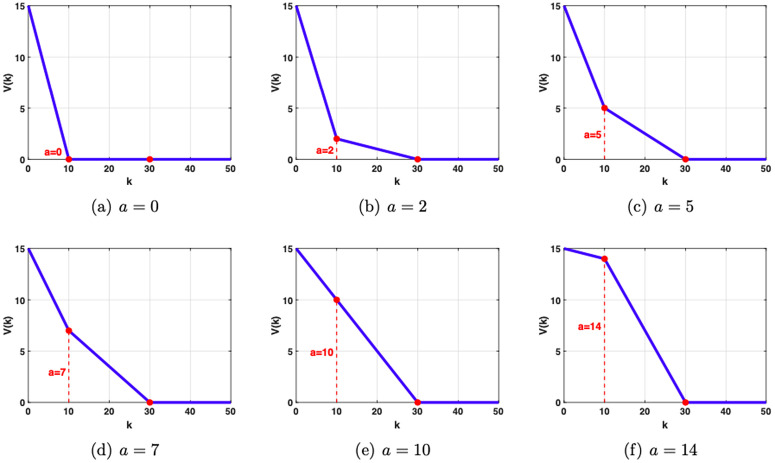
Idealized curves *V*(*k*) formed by 3 linear pieces, considering different values of the value *V*(10) = *a.* For any positive value *a* > 0, the elbow is clearly at $k^{*}=30$. the degree of certainty associated with this solution depends on the value of *a*: the smaller the value of *a* > 0, the stronger the evidence in favor of the solution $k^{*}=30$. For *a* = 0, the elbow is at $k^{*}=10$. For *a* > 10, the curve *V*(*k*) is not convex.

To illustrate the different principles employed by the proposed techniques, we consider several idealized scenarios based on artificial curves *V*(*k*) for which the location of the true solution (the elbow point) is exactly known. Moreover, these idealized scenarios were constructed so that the possible desired interval solutions could be readily inferred through simple visual inspection. Specifically, We assume that the curve *V*(*k*) is formed by three linear pieces (see Fig 4):


$$\begin{array}{c} V(k)=\left\{ \begin{array}{c} 15+\eta_{1}k,\;0\leq k<10,\\ b+\eta_{2}k,\;\;10\leq k<30,\\ 0\;\;\;\;30\leq k\leq 50. \end{array}\right. \end{array}$$ (15)

The parameters $\eta_{1}$, $\eta_{2}$ and *b* are chosen in order to have a continuous curve *V*(*k*) (at *k* = 10 and *k* = 30). To generate different scenarios, We used the value at *k* = 10 as the parameter of the experiment, namely *V*(10) = *a*. Choosing and fixing *a*, we have


$$\begin{array}{c} \eta_{1}=\frac{a-15}{10},\;\eta_{2}=-\frac{a}{20},\;b=\frac{3a}{2}. \end{array}$$ (16)

See Fig 4 for an example. More precisely, as depicted in Fig 4(a), if *a* = 0 the true solution/elbow is at $k^{*}=a$. Note that with *a* = 0, *V*(*k*) is formed by only two linear pieces. For any other positive value *a* > 0, the elbow is clearly at $k^{*}=30$. Moreover, if *a* > 10, the curve *V*(*k*) is not convex, as shown in Fig 4(f). Fig 4(e) provides the case with *a* = 10, and *V*(*k*) is again formed by only two linear pieces.

Note that the degree of certainty associated with this solution depends on the value of *a*: the smaller the value of *a* > 0, the stronger the evidence in favor of the solution $k^{*}=30$. The intervals provided by the proposed schemes are listed in Table 3. First, note that all the intervals (obtained with both schemes) always contain the true value of the elbow $k^{*}$. The results of the UAD-based intervals can be summarized as follows:

- with a=0⟹ the returned interval is $k_{1}^{*}=0$, $k_{2}^{*}=10$,
- with 0<a<10⟹ the returned interval is $k_{1}^{*}=10$, $k_{2}^{*}=30$,
- with 10≤a<30⟹ the returned interval is $k_{1}^{*}=0$, $k_{2}^{*}=30$.

**Table 3:** Intervals obtained with the proposed methods in the ideal scenarios of Section 5, for different values of *a.*

a	UAED-interval		SIC-interval	
	$k_{1}^{*}$	$k_{2}^{*}$	$k_{1}^{*}$ ($\ell_{1}=0.90$)	$k_{2}^{*}$ ($\ell_{2}=0.99$)
0	0	10	10	10
1	10	30	10	30
2	10	30	10	30
5	10	30	30	30
7	10	30	30	30
10	0	30	30	30
11	0	30	30	30
14	0	30	30	30

The SIC-based intervals can be summarized as follows:

- with a=0⟹ the returned interval is $k_{1}^{*}=10$, $k_{2}^{*}=10$,
- with 0<a≤2.5⟹ the returned interval is $k_{1}^{*}=10$, $k_{2}^{*}=30$,
- with 2.5<a<30⟹ the returned interval is $k_{1}^{*}=30$, $k_{2}^{*}=30$.

These results confirm that the UAD-based intervals are designed to maximize the probability $\text{Prob}\left(k^{*}\notin [0,k_{1}^{*})\text{ and }k^{*}\notin (k_{2}^{*},K]\right)$, whereas the SIC-based intervals are constructed with the simpler objective of ensuring that the true elbow $k^{*}$ lies within the interval.

## 6. Generalization of UAED and SIC for non-uniform sampled and continuous-valued parameter $z_{k}$

In the previous section, we have considered a uniform discrete index *k* = 0,1,2,...,*K*, is a discrete variable (with increasing step 1) that represents the number of components/parameters in the analyzed model. Thus, the error curve *V*(*k*) was (generally a non-increasing) function defined a discrete domain with a uniform sampling *k* = 0,1,2,...,*K* with step 1. In other frameworks (see an example below in Section 6.2), we can have an error curve V(z):ℝ→ℝ that is a function of a continuous parameter z∈ℝ. However, we can observe only some points of this curve that are non-uniformly sampled at $z_{0}<z_{1}<z_{2}<...<z_{K}$. Thus we finally observe *V*(*z*) in a finite number of points, $V(z_{1}),V(z_{2}),...V(z_{K})$ and we assume, without loosing generality, $V(z_{k})=0$. Recall that generally the difference $z_{k+1}-z_{k}$ varies with *k*, and generally $|z_{k+1}-z_{k}|\text{≠}1$, but we always have $z_{k+1}-z_{k}>0$.

### 6.1. Extended algorithms

In the following, we assume *V*(*z*) is a non-increasing function so that $V(z_{0})\geq V(z_{1})\geq V(z_{2})\geq ...\geq V(z_{K})$.

**The UAED solutions.** Let define


$$\begin{array}{c} k_{1}\in \{z_{0},z_{1},...,z_{K}\},\;k_{2}\in \{z_{0},z_{1},...,z_{K}\},\text{ and }k_{1}\leq k_{2}, \end{array}$$

Therefore, the two optimal points forming the interval solution are denoted as $\left[k_{1}^{*},k_{2}^{*}\right]$. Adapting the UAED solutions for this new framework is possible changing the definitions of the areas:


$$\begin{array}{cc} A_{1} & =\frac{\left(V(z_{0})+V(k_{1}))(k_{1}-z_{0}\right)}{2},\\ A_{2} & =\frac{\left(V(k_{1})+V(k_{2}))(k_{2}-k_{1}\right)}{2},\;\text{ and }\\ A_{3} & =\frac{V(k_{2})(z_{K}-k_{2})}{2}, \end{array}$$ (17)

and the final solution will given by


$$[k_{1}^{*},k_{2}^{*}]=\text{arg}\min_{k_{1},k_{2}}\{A_{1}+A_{2}+A_{3}\},\;k_{1}<k_{2},$$

where *A*_1_, *A*_2_ and *A*_3_ are defined in Eq. (17).

**The SIC solutions.** Considering the set of *z* values $\left\{z_{0},z_{1},...,z_{K}\right\}$ where *V*(*z*) is evaluated, we have a one-to-one (bijective) correspondence/mapping $k⟷z_{k}$, i.e., *k* = 0 corresponds to *z*_0_, *k* = 1 corresponds to *z*_1_, and so on. Furthermore, we can rewrite the penalized minimization problem as


$$\begin{array}{c} C(z_{k},\lambda )=V(z_{k})+\lambda z_{k},\;\lambda >0,\text{ and }z_{k}\in \{z_{0},z_{1},...,z_{K}\}, \end{array}$$ (18)

where varying λ the position of the minimum changes. We can still refer to the optimal index $k^{*}⟷z_{k^{*}}$, i.e.,


$$\begin{array}{c} k^{*}(\lambda )=\text{arg}\min_{k}C(z_{k},\lambda ), \end{array}$$ (19)

where again $k^{*}(\lambda ):[0,\lambda_{\text{max}}]\to \{0,1,2...,K\}$. We need also to change the procedure to obtain $\lambda_{\text{max}}$, i.e.,


$$\begin{array}{c} \lambda_{\text{max}}=\max\left[\frac{V(z_{0})-V(z_{i})}{z_{i}-z_{0}}\right],\;\text{for }i=1,...,\text{K}. \end{array}$$ (20)

Recall that we have $V(z_{0})\geq V(z_{i})$ and $z_{i}\geq z_{0}$, by assumption. Thus, for $\lambda \geq \lambda_{\text{max}}$ the minimum is reached at *z*_0_ the $k^{*}=\text{arg}\min_{k}C(z_{k},\lambda )=0$. Hence, $k^{*}(\lambda )$ is still a non-increasing, piecewise constant function that takes discrete values from 0 to *K*, where $k^{*}(0)=K$ and $k^{*}(\lambda )=0$ for $\lambda \geq \lambda_{\text{max}}$. Therefore, the rest of the algorithm remained unchanged.

### 6.2. Example of application: selection of the regularization parameter

For the sake of simplicity, in order to provide an example of application, We focus on a linear regularized regression problem. Identical considerations can be made for a nonlinear scenario or a classification setting. Consider the observed dataset $\{x_{i},y_{i}{\}}_{i=1}^{N}$ where the $x_{i}=[x_{i,1},...,x_{i,R}{]}^{⊤}$ are input vectors, $X=[x_{1},...,x_{N}]$ is an R×N input matrix, and $y=[y_{1},...,y_{N}{]}^{⊤}$ is the corresponding output vector. We also define the vector of unknown parameters $\beta =[\beta_{1},...,\beta_{R}]$, that can be estimated by the following minimization,


$$\begin{array}{c} \beta_{\gamma }^{*}=\text{arg}\min_{\beta }[||y-\beta X|{|}^{2}+\gamma ||\beta |{|}^{\alpha }],\;\gamma >0,\;\alpha \geq 1. \end{array}$$ (21)

The second term $\gamma ||\beta |{|}^{\alpha }$ is a regularizer, employed for (a) avoiding overfitting, (b) numerical issues, and (c) forcing some characteristics in the final solution (as sparsity in the vector $\beta^{*}$, when α=1). The scenario corresponding to α=2 is known as ridge regression, whereas the scenario corresponding to α=1 is the LASSO regression [44,45]. The positive continuous parameter γ controls the strength of the regularization term.

The first term above is the well-known mean square error (MSE), that is, $\text{MSE}=||y-\beta X|{|}^{2}$. Hence, for each possible γ, we have an optimal $\beta_{\gamma }^{*}$ and a corresponding value of MSE, i.e.,


$$\begin{array}{c} \text{MSE}(\gamma )=||y-\beta_{\gamma }^{*}\;X|{|}^{2}. \end{array}$$ (22)

Clearly, with γ=0, we have the smallest MSE and if $0<\gamma_{1}<\gamma_{2}$ we have $\text{MSE}(\gamma_{1})<\text{MSE}(\gamma_{2})$, i.e., $\text{MSE}(0)<\text{MSE}(\gamma_{1})<\text{MSE}(\gamma_{2})$. The optimal selection of γ, which balances model fitting, generalization, and other desirable properties, remains an active area of research [44,45]. Note that if we set z=1/γ and define


$$\begin{array}{c} V(z)=\text{MSE}(z)=\text{MSE}(1/\gamma ), \end{array}$$ (23)

we have a non-increasing error function of *z*. Another possible definition (similar to other information criteria) is V(z)=log(MSE(z)). Therefore, we can apply UAED and SIC to provide an optimal choice of $z^{*}=1/\gamma^{*}$, and also an interval solution $\left[k_{1}^{*},k_{2}^{*}\right]$ with $z^{*}\in [k_{1}^{*},k_{2}^{*}]$, in order to quantify the uncertainty in the selection (as described in the previous sections).

## 7. Numerical experiments

In this section, we evaluate the proposed construction of the intervals across different settings, including experiments with artificial data (Sections 7.1 and 7.3), a synthetic curve *V*(*k*) defined analytically (Section 7.2), and two real-world datasets are analyzed in Sections 7.4–7.5. An application for selecting a LASSO-shrinkage parameter, applying the extensions presented in Section 6, is provided in Section 7.6. The MATLAB code used for the experiments is also made available in https://zenodo.org/records/21639880.

### 7.1. Order selection in an auto-regressive model with a Laplacian noise

We generate a dataset of *T* pairs $\{t,y_{t}{\}}_{t=1}^{T}$, where *t* is an integer temporal index and the signal $y_{t}$ is a scalar value for each *t*. We consider the following auto-regressive model,


$$\begin{array}{c} y_{t}=\theta_{1}y_{t-1}+\theta_{2}y_{t-2}+...\theta_{k}y_{t-k}+\epsilon_{t},\;\text{for }t=1,...,\text{T}, \end{array}$$ (24)

where $\theta_{k}=[\theta_{1},\theta_{2},...,\theta_{k}{]}^{⊤}$. We assume that $\epsilon_{t}$ is a Laplacian noise, i.e.,


$$\begin{array}{c} p(\epsilon_{t})=\frac{1}{2b}\exp\left(-\frac{\left|\epsilon_{t}-\mu \right|}{b}\right), \end{array}$$

with zero mean, μ=0, and variance $\sigma_{\epsilon }^{2}=2b^{2}$. The goal is to infer the true order of the model $k_{\text{true}}$. This problem is very frequent in signal processing, statistics, and machine learning [10,11]. Note that it is possible to easily generate random samples from a Laplace density [50], Chapter 2]. Therefore, starting with null initial conditions, it is possible to generate data according to the model in Eq. (24). We test two levels of noise $\sigma_{\epsilon }=0.5$, i.e., *b* = 0.35, with standard deviation $\sigma_{\epsilon }=5$, i.e., *b* = 3.53. We also consider different numbers of data, T∈{200,2000}.

In this example, we test three possible values of the order of the model, $k_{\text{true}}\in \{3,5,7\}$, where we have employed the following formula for the coefficients: $\theta_{i}=(-1{)}^{i-1}\exp\{-0.3(i-1)\}$, to ensure that the system in Eq. (24) is stable. More specifically, we have


$$\begin{array}{cc} k_{\text{true}} & =3\;\;⟹\;\theta_{1}=1,\;\theta_{2}=-0.7408,\;\theta_{3}=0.5488;\\ k_{\text{true}} & =5\;\;⟹\;\theta_{1}=1,\;\theta_{2}=-0.7408,\;\theta_{3}=0.5488,\;\theta_{4}=-0.4066,\;\theta_{5}=0.3012;\\ k_{\text{true}} & =7\;\;⟹\;\theta_{1}=1,\;\theta_{2}=-0.7408,\;\theta_{3}=0.5488,\;\theta_{4}=-0.4066,\;\theta_{5}=0.3012,\\ & \;\;\;\;\;\;\;\;\;\;\;\;\;\;\;\theta_{6}=-0.2231,\;\theta_{7}=0.1653. \end{array}$$

Note that the last coefficients become smaller, making their detection/estimation more difficult, especially with $\sigma_{\epsilon }=5$. Hence, the scenario with $k_{\text{true}}=7$ is more difficult in terms of estimating the order of the model, especially with a high noise power.

**Fig 5:**
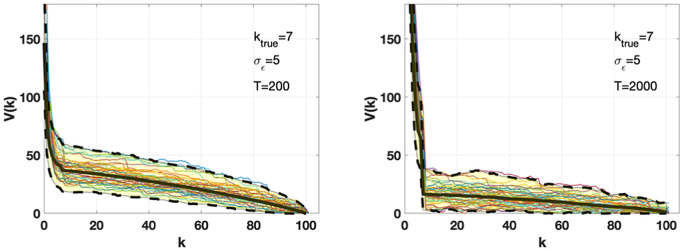
Examples of the curves *V*(*k*) of Section 7.1 in 50 different runs for $k_{true}=7$, $\sigma_{\epsilon }=5$ and T∈{200,2000}; the median curve is depicted with a black solid line, and the yellow area between two black dashed lines represents the 98% of the empirical distribution provided by the 10^3^ runs.

Given each combination of the values of $\sigma_{\epsilon }$, *T*, and $k_{\text{true}}$, we generated the data $y=[y_{1},...,y_{T}]$ according to the model (24). In all scenarios, we averaged the results with 10^3^ independent runs, generating a new time series of *T* for each run. Moreover, in all the simulations, we consider $V(k)=-2\log(\ell_{\text{max}})$ with $\ell_{\text{max}}=\max_{\theta }p(y|\theta_{k})$ with k≤K (setting *K* = 100), where $p(y|\theta_{k})$ is induced by Eq. (24), in order to allow the comparison with other schemes in the literature, as shown in Tables 1 and 5. Note that, because we consider Laplacian noise, $\ell_{\text{max}}$ is not provided in an analytic form. Indeed, it requires the knowledge of the maximum likelihood estimator ${\hat{\theta }}_{k}=\text{arg}\max_{\theta }p(y|\theta_{k})$, then $\ell_{\text{max}}=p(y|{\hat{\theta }}_{k})$. We consider the least-squares estimation of vector $\theta_{k}$ as an approximation of ${\hat{\theta }}_{k}$. Fig 5 shows 50 examples of the curves *V*(*k*) in different runs (for $k_{\text{true}}=7$, $\sigma_{\epsilon }=5$ and T∈{200,2000}), jointly with the median curve (black solid line) and a yellow area between two black dashed lines, corresponding to the 98% of the empirical distribution provided by the 10^3^ runs.

In Table 4, we provide the results of different information criteria: BIC [47], AIC [48], HQIC [36], and UAED [29]. The best results are marked with yellow cells. We can observe that BIC and UAED provide the best results in terms of the correct decision rate $p_{A}\in [0,1]$, inferring the order of the model (i.e., the times that the method selects the correct order over the total number of simulations). Recall that the first proposed interval method is designed to extend the derivation of the UAED. Table 5 shows the median intervals (obtained using the two methods introduced in the previous section) over the 10^3^ runs in the different scenarios. Moreover, The table gives the rate $p_{\text{in}}\in [0,1]$ of the intervals containing $k_{\text{true}}$ (clearly, including the extreme values of the interval). Namely, $p_{\text{in}}$ is the ratio of the number of times $k_{\text{true}}$ is inside the constructed interval over the total number of runs. This value $p_{\text{in}}$ plays the same role as the quantity 1−α where α is the confidence level in classical interval estimation. We observed that both achieved excellent performance. The best results in terms of shorter intervals and $p_{\text{in}}$ are marked with a green cell. Both methods appear to be virtually insensitive to the noise power. The geometric-based approach always obtains good results, even with a small amount of data. Whereas the SIC-based approach seems to work better with a larger number of data points, suffering more in the case of smaller data points (designing much longer intervals in these cases). This can be clearly seen by looking at the median lengths $L_{\text{med}}$ of the intervals:


$$\begin{array}{cc} & \mathrm{Geometric-based}⟹L_{\text{med}}\in \{9,2,11,2,4,4,4,4,6,5,6,5\}\\ & \mathrm{SIC-based}\;\;\;\;⟹L_{\text{med}}\in \{6,0,8,0,85,0,77,0,90,4,90,4\}. \end{array}$$

**Table 4:** Summary of the correct-decision rate $p_{A}\in [0,1]$ (averaged over 10^3^ runs) for the IC methods, in the experiment of Section 7.1.

Scenario			Method			
$k_{true}$	$\sigma_{\epsilon }$	*T*	UAED	BIC	AIC	HQIC
3	0.5	200	$p_{A}\approx 0.94$	$p_{A}\approx 0.97$	$p_{A}\approx 0.79$	$p_{A}\approx 0.73$
		2000	$p_{A}=1$	$p_{A}=1$	$p_{A}\approx 0.88$	$p_{A}\approx 0.89$
	5	200	$p_{A}\approx 0.96$	$p_{A}\approx 0.99$	$p_{A}\approx 0.78$	$p_{A}\approx 0.71$
		2000	$p_{A}=1$	$p_{A}\approx 0.99$	$p_{A}\approx 0.89$	$p_{A}\approx 0.90$
5	0.5	200	$p_{A}\approx 0.89$	$p_{A}=0.95$	$p_{A}\approx 0.78$	$p_{A}\approx 0.72$
		2000	$p_{A}=1$	$p_{A}\approx 0.99$	$p_{A}\approx 0.84$	$p_{A}\approx 0.84$
	5	200	$p_{A}\approx 0.89$	$p_{A}\approx 0.97$	$p_{A}\approx 0.77$	$p_{A}\approx 0.68$
		2000	$p_{A}=1$	$p_{A}\approx 0.99$	$p_{A}\approx 0.83$	$p_{A}\approx 0.83$
7	0.5	200	$p_{A}\approx 0.58$	$p_{A}\approx 0.30$	$p_{A}\approx 0.60$	$p_{A}\approx 0.56$
		2000	$p_{A}=1$	$p_{A}\approx 0.98$	$p_{A}\approx 0.79$	$p_{A}\approx 0.81$
	5	200	$p_{A}\approx 0.58$	$p_{A}\approx 0.30$	$p_{A}\approx 0.58$	$p_{A}\approx 0.54$
		2000	$p_{A}=1$	$p_{A}\approx 0.99$	$p_{A}\approx 0.78$	$p_{A}\approx 0.78$

**Table 5:** Performance of the designed intervals in Section 7.1. We provide (a) the median interval $I_{med}$ over all runs and (b) the rate $p_{in}\in [0,1]$ of the intervals containing $k_{true}$ (clearly, including the extremes of the interval). This value $p_{in}$ plays the same role of the quantity 1−α where α is the confidence level (in classical interval estimation).

Scenario			Method	
$k_{true}$	$\sigma_{\epsilon }$	*T*	Geometric-based	SIC-based
3	0.5	200	$I_{\text{med}}=[3,12]$	$I_{\text{med}}=[3,9]$
			$p_{\text{in}}\approx 0.997$	$p_{\text{in}}=1$
		2000	$I_{\text{med}}=[1,3]$	$I_{\text{med}}=[3,3]$
			$p_{\text{in}}=1$	$p_{\text{in}}=1$
	5	200	$I_{\text{med}}=[3,14]$	$I_{\text{med}}=[3,11]$
			$p_{\text{in}}\approx 0.995$	$p_{\text{in}}=1$
		2000	$I_{\text{med}}=[1,3]$	$I_{\text{med}}=[3,3]$
			$p_{\text{in}}=1$	$p_{\text{in}}=1$
5	0.5	200	$I_{\text{med}}=[2,6]$	$I_{\text{med}}=[5,90]$
			$p_{\text{in}}\approx 0.995$	$p_{\text{in}}\approx 1$
		2000	$I_{\text{med}}=[1,5]$	$I_{\text{med}}=[5,5]$
			$p_{\text{in}}=1$	$p_{\text{in}}=1$
	5	200	$I_{\text{med}}=[2,6]$	$I_{\text{med}}=[5,82]$
			$p_{\text{in}}\approx 0.992$	$p_{\text{in}}=1$
		2000	$I_{\text{med}}=[1,5]$	$I_{\text{med}}=[5,5]$
			$p_{\text{in}}=1$	$p_{\text{in}}=1$
7	0.5	200	$I_{\text{med}}=[2,8]$	$I_{\text{med}}=[3,93]$
			$p_{\text{in}}\approx 0.935$	$p_{\text{in}}\approx 0.983$
		2000	$I_{\text{med}}=[2,7]$	$I_{\text{med}}=[3,7]$
			$p_{\text{in}}=1$	$p_{\text{in}}=1$
	5	200	$I_{\text{med}}=[2,8]$	$I_{\text{med}}=[3,93]$
			$p_{\text{in}}\approx 0.927$	$p_{\text{in}}\approx 0.970$
		2000	$I_{\text{med}}=[2,7]$	$I_{\text{med}}=[3,7]$
			$p_{\text{in}}=1$	$p_{\text{in}}=1$

The SIC approach is able to return intervals with zero median lengths (maximum certainty) when *T* = 2000 (larger number of data points) and $k_{\text{true}}=3$ and $k_{\text{true}}=5$, but struggles when *T* = 200 where the median lengths are quite large, such as 85, 77, 90 and 90, compared with the median lengths obtained with the geometric approach, respectively 4, 4, 6 and 6. In this sense, the geometric approach appears to be more robust.

### 7.2. Artificial curve *V*(*k*) with known analytic form

In this section, we consider a synthetic error curve *V*(*k*) with a known analytic form. In this way, we can observe some convergence properties of the different schemes and the ability to encode the geometric invariant features of a curve *V*(*k*). More specifically, the goal of this experiment is to analyze the behaviors of the elbow detectors [29–32], the index of effective number of variables (ENV) recently introduced in [38], and the two constructions of intervals proposed in this work, the geometric-based and the SIC-based approaches. We consider the function


$$\begin{array}{c} V^{\prime }(k)=e^{-0.1k},\;k=0,1,2...,K, \end{array}$$

and study different values of *K*. For each possible value of $K\in \{20,50,500,5000,10^{4}\}$, we define $V(k)=V^{\prime }(k)-\min V^{\prime }(k)=e^{-0.1k}-e^{-0.1K}$, such that *V*(*K*)=0 in any case. We tested the elbow detectors [29–32], the ENV index, and the construction of the intervals, obtaining the results given in Table 6.

**Table 6:** Results in the synthetic experiment of Section 7.2.

Methods	*K* = 20	*K* = 50	*K* = 500	*K* = 5000	*K* = 10^4^
Elbow detectors	8	16	39	62	69
ENV index	13.756	19.338	20.016	20.016	20.016
Geometric-based interval	[5,12]	[10,24]	[17,56]	[21,83]	[22,91]
SIC-based interval	[20,20]	[30,50]	[30,53]	[30,54]	[30,54]

We can observe that the detected elbows are always contained in the geometric-based intervals, as expected and stated in Section 3. However, the geometric-based intervals present an undesirable dependence on *K*, particularly in the second extreme of the interval. On the other hand, the ENV index converges to the value ${\bar{I}}_{\text{ENV}}=20.016$ as *K* increases, as expected [38]. The SIC-based intervals present the same stability property converging to the interval [30] as *K* increases.

### 7.3. Choice of the number of clusters

Let us consider a mixture of five bidimensional Gaussian distributions $𝒩\left(\mu_{i},\Sigma_{i}\right)$ where:

- $\mu_{1}=[3,0]$, $\Sigma_{1}=[0.3,0;0,2]$,
- $\mu_{2}=[14,5]$, $\Sigma_{2}=[1.5,0.7;0.7,1.5]$,
- $\mu_{3}=[-5,-10]$, $\Sigma_{3}=[1.5,0.7;0.7,1.5]$,
- $\mu_{4}=[10,-10]$, $\Sigma_{4}=[1.5,0;0,1.5];$,
- and $\mu_{5}=[-5,5]$, $\Sigma_{5}=[1,-0.8;-0.8,1]$.

We generated 2500 simulated datasets from this mixture. Thus, the generated data form 5 disjoint clusters. In this experiment, we define $V(k)=\log\left[\sum_{j=1}^{k+1}\mathrm{var}(j)\right]$, where var(j) represents the inner variance of the *j*-th cluster. Each value of var(j) was averaged over 200 runs, applying a k-means algorithm to define the memberships of each cluster at each run. The case *k* = 0 corresponds to a unique single cluster formed by all data. Hence, the total number of clusters is given by *k* + 1. We assume *K* = 50 as the maximum number of possible clusters. Note that With this choice of *V*(*k*), we can apply only UAED, whereas the rest of the IC in Table 1 cannot be applied. In this experiment, UAED suggested the right number of clusters, 5. The geometric-based interval in this example is [2,6], whereas the SIC-based interval is [5,6]. Both contain the correct number of clusters, and the SIC-based interval is shorter.

### 7.4. Feature selection in a soundscape emotion real dataset

In this section, we address the variable selection problem in a regression setting using real-world data, specifically focusing on a soundscape emotion dataset. More specifically, a dataset of *N* pairs $\{x_{n},y_{n}{\}}_{n=1}^{N}$ is given, where each input vector $x_{n}=[x_{n,1},...,x_{n,K}]$ is formed by *K* variables, and the outputs $y_{n}$’s are scalar values. We assume K≤N and a linear measurement model,


$$\begin{array}{c} y_{n}=\theta_{0}+\theta_{1}x_{n,1}+\theta_{2}x_{n,2}+...\theta_{K}x_{n,K}+\epsilon_{n}, \end{array}$$ (25)

where $\epsilon_{n}$ is a Gaussian perturbation with zero mean and variance $\sigma_{\epsilon }^{2}$, i.e., $\epsilon_{n}\sim 𝒩(\epsilon |0,\sigma_{\epsilon }^{2})$. In the soundscape emotion dataset analyzed for instance in [12], there are *K* = 122 features and *N* = 1214 number of data points. The output represents a variable defined as “arousal” in [12].

After ranking the 122 variables as suggested in [12], We set again $V(k)=-2\log(\ell_{\text{max}})$ where $\ell_{\text{max}}=\max_{\theta }p(y|\theta_{k})$ with k≤K, and where the likelihood function $p(y|\theta_{k})$ is induced by Eq. (25). This choice of *V*(*k*) allows the computation of AIC, BIC, and HQIC (see Table 1). The results of the different IC and the intervals built by the proposed schemes are given below:

- BIC suggests a model with 17 variables.
- AIC chooses 44 variables.
- HQIC selects a model with 41 variables.
- UAED suggests a model with 11 variables.
- The interval built with the geometric procedure is [7,41].
- The interval based on the SIC procedure is [7,25].

**Fig 6:**
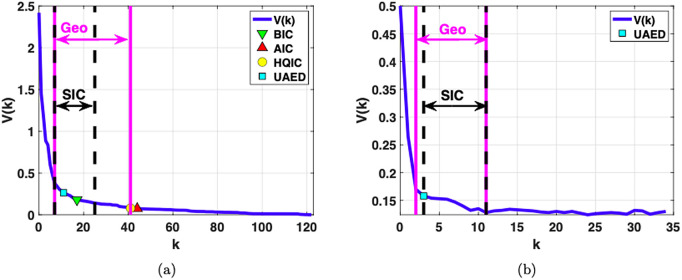
V(*k*) curves and results for the experiments in (a) Section 7.4 and (b) Section 7.5 with real data.

Note that the geometric-based interval contains all the IC, except for the result of AIC (44 variables). The SIC-based interval also excludes the solution provided by HQIC (41 variables). In this experiment, the SIC-based interval was shorter than the geometric-based interval. Both intervals are in line with other previous studies regarding this dataset and with the experts’ recommendations in the literature, e.g., [12]. Fig 6(a) shows the corresponding curve *V*(*k*) and summarizes all the results provided by the IC and obtained intervals.

### 7.5. Feature selection in a classification problem with a nonalcoholic fatty liver disease real dataset

In this section, we consider an example of biomedical applications, which are nowadays extremely important in signal processing and machine learning [51,52]. In [53], the authors study the most important variables for predicting patients at risk of developing non-alcoholic fatty liver disease. The dataset contains 1525 patients. The authors of [53] employed a random forest (RF) algorithm as a classifier and ranked the input variables, selecting the most relevant ones. The resulting 4 most important features are according to this raking: (a) insulin resistance, (b) ferritin, (c) serum insulin levels, and (d) triglycerides. The authors in [53] employed cross-validation (CV) to find the optimal number of features (that is 4), and this result was supported by expert opinions.

In this section, we define V(k)=1−accuracy(k) as the error curve, as shown in Fig 6(b), using accuracy(k) obtained in [53] and after ranking the 35 variables as in [53]. Note that *V*(0)=0.5 representing a completely random binary classification. Note that, even with this choice of the curve V(k)=1−accuracy(k), we can still apply UAED and SIC, as shown in Table 1. Other information criteria cannot be applied in this context. However, we can obtain the intervals based on the geometric approach (which is related to the UAED derivation) and the SIC approach. As shown in Fig 6(b), the resulting geometric interval is [2,11] and the SIC-based interval is [3,11]. Both contain 4 variables, which is exactly the result provided in [53], obtained by applying a cross-validation approach and supported by the experts’ opinions.

### 7.6. Selection of the the LASSO-shrinkage parameter

Consider a dataset $\{x_{i},y_{i}{\}}_{i=1}^{N}$ where the $x_{i}=[x_{i,1},...,x_{i,R}{]}^{⊤}$, $X=[x_{1},...,x_{N}]$ are input vectors and R×N input matrix, whereas $y=[y_{1},...,y_{N}{]}^{⊤}$ is the output vector. We also define the vector of unknown parameters $\beta =[\beta_{1},...,\beta_{R}{]}^{⊤}$, and assume the observation model


$$\begin{array}{c} y=\beta X+\epsilon , \end{array}$$

where ϵ is a noise perturbation that we consider Gaussian with zero mean and variance $\sigma^{2}=1$. We set *R* = 30 and generate artificially *N* = 200 data pairs following model considering $x_{i,r}\sim 𝒩(x|0,25)$ (randomly drawn for each *i*, *r* at each run), and assuming the vector


$$\begin{array}{c} \beta_{\text{true}}=[\underset{20}{\underbrace{1,1,1,1,..,1}}\underset{10}{\underbrace{0,0,0,0,..,0}}{]}^{⊤}, \end{array}$$

Hence, the last 10 features do not affect the outputs $y_{i}$. After data generation, we apply a LASSO techniques [44,45] to obtain and estimate the coefficient vector $\beta_{\gamma }^{*}$. Namely, we follow the minimization of a square error with an L_1_ regularizer,


$$\begin{array}{c} \beta_{\gamma }^{*}=\text{arg}\min_{\beta }[||y-\beta X|{|}^{2}+\gamma ||\beta |{|}_{1}],\;\gamma >0. \end{array}$$ (26)

We use a thin grid of γ values: as γ increases, the number of zeros in $\beta_{\gamma }^{*}$, until a maximum value of γ sun that $\beta_{\gamma }^{*}$ has only null entries.

**Fig 7:**
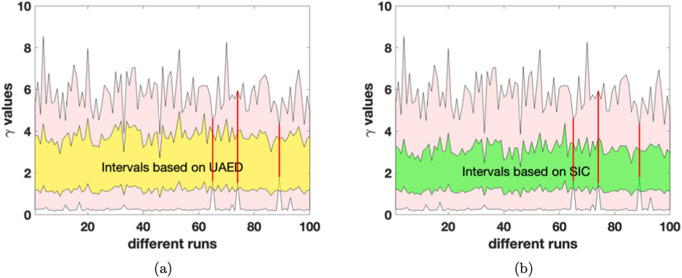
The groundtruth intervals in each realization $\left[\gamma_{1}^{*},\gamma_{2}^{*}\right]$ are depicted with pink shaded areas. In these intervals, the γ values provide exactly 10 zeros (well-located) in $\beta_{\gamma }^{*}$. **(a)** The intervals provided by the UAED are shown in yellow shaded areas.The only three intervals – shown with red vertical segments – that are not completely contained in the yellow area are: [1.78,4.34], [1.70,5.82], and [1.80,4.19]. In all cases, the UAED-based intervals had a slightly smaller first element (1.57, 1.58, and 1.56, respectively). **(b)** The intervals provided by the SIC are shown in green shaded areas. The only three intervals – shown with red vertical segments – that are not completely contained in the green area are [1.81,4.26], [1.80,5.75], and [1.83,4.23]. In all cases, the SIC-based intervals have a slightly smaller first element (1.63, 1.65 and 1.60, respectively).

We denote as $\gamma_{1}^{*}$ the smallest value of γ such that we have only 10 zeros (located at the last ten positions of $\beta_{\gamma }^{*}$) and denote s $\gamma_{2}^{*}$ the biggest value of γ such that we have only 10 zeros (located at the last ten positions of $\beta_{\gamma }^{*}$). Hence, a reasonable choice of γ for our problem is any γ inside $\left[\gamma_{1}^{*},\gamma_{2}^{*}\right]$. For each realization of the data, the groundtruth interval $\left[\gamma_{1}^{*},\gamma_{2}^{*}\right]$ is shown in Fig 7 with pink shaded areas. We apply the procedures in Sections 6 a 6.2 to find the interval solutions based on the UAED and SIC. We can observe in Fig 7, all the γ values suggested by the intervals based UAED and SIC are completely contained in the groundtruth intervals $\left[\gamma_{1}^{*},\gamma_{2}^{*}\right]$, with the exception of 3 runs (over a total number of 100 independent runs), where a small portions of the first parts of the intervals are slight out the groundtruth intervals (that, in these 3 scenarios, are highlighted with solid red lines). Hence, it appears clear that the interval solutions provided by UAED and SIC contain safe and useful γ values to use in a LASSO regression problem.

## 8. Conclusions

In this study, we proposed two alternative constructions for deriving intervals that capture the uncertainty associated with determining the number of components in nested models. We have also extended the proposed techniques to cases where the error curve V(z):ℝ→ℝ, defined over a continuous parameter z∈ℝ, is observed only at non-uniformly sampled points. This extension enables their application for selecting shrinkage regularization parameters.

The proposed approaches do not require knowledge of a likelihood function, making them broadly applicable across various domains, including regression and classification, feature and/or order selection, clustering, change-point detection, and dimensionality reduction, among others. We have also extensively discussed the connection between our methods and widely used information criteria in the literature. Additionally, a MATLAB code is provided to facilitate the adoption of these methods by researchers and practitioners in applied settings.

Extensive experiments on both synthetic and real data demonstrated the strong performance of the proposed schemes. In particular, the results highlight the following:

- Both procedures can be applied for selecting the shrinkage parameters in classification or regression problems with regularizations.
- Both procedures design intervals that contain the true number of components (or the number of components suggested by the experts) in more than 90% of the runs/realizations.
- The geometry-based procedure appears to be more robust to variations in the number of data points; however, its performance is influenced by the total number of components *K*.
- The SIC-based procedure tends to yield the most accurate results as the number of data points increases. Conversely, when the data size is limited, this approach often produces relatively wide intervals. A notable property of the SIC-based method is the convergence and invariance of the resulting interval as K→∞.
- Both approaches seems to exhibit a notable degree of robustness to the noise levels affecting the data.

The proposed schemes provide particularly suitable solutions when the error curve *V*(*k*) tends to be convex, as evidenced by the final two experiments involving real datasets and supported by our practical experience. It is also important to highlight that the proposed methods can be applied in the absence of a likelihood function. Consequently, we argue that both procedures serve as (a) universal, (b) automatic, and (c) computationally efficient tools for quantifying the uncertainty inherent in model selection problems without the need for resampling techniques or cross-validation schemes. As a future research direction, the problem of identifying the elbow point in a noisy error curve *V*(*k*), that is, considering uncertainty in the evaluations of *V*(*k*), appears particularly interesting and deserves further investigation. Distributed scenarios, such as federated learning frameworks designed to preserve privacy, can also be investigated. In such settings, each local node processes its own data and produces a corresponding error curve *V*(*k*), thereby introducing an additional source of uncertainty at the central aggregation node.
